# Semantically Transparent and Opaque Compounds in German Noun-Phrase Production: Evidence for Morphemes in Speaking

**DOI:** 10.3389/fpsyg.2016.01943

**Published:** 2016-12-27

**Authors:** Antje Lorenz, Pienie Zwitserlood

**Affiliations:** ^1^Department of Psychology, Neurocognitive Psychology, Humboldt-Universität zu BerlinBerlin, Germany; ^2^Department of Psychology, Psycholinguistics and Cognitive Neuroscience, University of MünsterMünster, Germany

**Keywords:** compound nouns, semantic transparency, morphology, gender congruency, speech production, picture-word task

## Abstract

This study examines the lexical representation and processing of noun-noun compounds and their grammatical gender during speech production in German, a language that codes for grammatical gender (masculine, feminine, and neuter). Using a picture-word interference paradigm, participants produced determiner-compound noun phrases in response to pictures, while ignoring written distractor words. Compound targets were either semantically transparent (e.g., birdhouse) or opaque (e.g., hotdog), and their constituent nouns either had the same or a different gender (internal gender match). Effects of gender-congruent but otherwise unrelated distractor nouns, and of two morphologically related distractors corresponding to the first or second constituent were assessed relative to a completely unrelated, gender-incongruent distractor baseline. Both constituent distractors strongly facilitated compound naming, and these effects were independent of the targets' semantic transparency. This supports retrieval of constituent morphemes for semantically transparent and opaque compounds during speech production. Furthermore, gender congruency between compounds and distractors did not speed up naming in general, but interacted with gender match of the compounds' constituent nouns, and their semantic transparency. A significant gender-congruency effect was obtained with semantically transparent compounds, consisting of two constituent nouns of the same gender, only. In principle, this pattern is compatible with a multiple lemma representation account for semantically transparent, but not for opaque compounds. The data also fit with a more parsimonious, holistic representation for all compounds at the lemma level, when differences in co-activation patterns for semantically transparent and opaque compounds are considered.

## Introduction

It is still a matter of debate whether the morphological structure of polymorphemic words, such as compounds (e.g., sunflower), determines their lexical representation and processing, and if so, how. While network theories assume that morphology is not explicitly represented in the lexical system (Plaut and Gonnerman, 2000; Baayen et al., 2013), others argue that morphological structure does play a role. Full-listing models predict holistic processes for familiar complex words, and morpheme-based processes for novel complex words only (Butterworth, 1983), whereas full-decomposition models predict morpheme-based processes for novel and familiar complex words alike (Taft and Forster, 1976). Furthermore, dual- or multiple-route accounts assume that the type of processing depends on different characteristics of complex words, such as their frequency or semantic transparency (e.g., Zwitserlood, 1994; Schreuder and Baayen, 1995; Kuperman et al., 2009; Marelli and Luzzatti, 2012; Xu and Taft, 2015). Semantic transparency refers to the meaning relation between the complex word and its constituents. Dual-route accounts assume morpheme-based processing for semantically transparent complex words (e.g., birdhouse), but (more) holistic processing for opaque words (e.g., hotdog). Most data that test such predictions come from comprehension studies, or from studies on the interface between comprehension and production, presenting complex words as distractors to pictures with monomorphemic names (e.g., Zwitserlood et al., 2002; Köster and Schiller, 2008; Lüttmann et al., 2011b; Verdonschot et al., 2012). To date, studies on the actual production of complex words are quite rare (e.g., Roelofs and Baayen, 2002; Lüttmann et al., 2011a; Jacobs and Dell, 2014).

Our study focuses on the production of semantically transparent and opaque compounds in German, a morphologically rich language with many compounds. German also codes for grammatical gender, with three gender classes (masculine, feminine, and neuter) that are overtly marked on the definite determiners of nouns: *der*_*masc*_*, die*_*fem*_, and *das*_*neut*_ [the]. Though phonological and semantic regularities exist that highly correlate with gender in German (e.g., Köpcke and Zubin, 1984; Schwichtenberg and Schiller, 2004; Zubin and Köpcke, 2009), gender is not fully predictable by such features. Therefore, a word's gender is assumed to be stored in the mental lexicon, as an inherent property of the lexical specification of nouns (see Levelt, 1989; Schriefers and Jescheniak, 1999). As in English, the rightmost constituent of German compounds is the morphological/morpho-syntactic head, which determines syntactic features including grammatical gender (e.g., Williams, 1981). While *Haus* has neuter gender, the gender of the modifier, the first constituent (*Vogel*) is masculine. The constituents thus differ or mismatch in gender, but note that this is irrelevant for gender specification of the whole word ([*Vogel*_*masc*_*haus*_*neut*_]_*neut*_). In contrast, in a compound such as *Tee*_*masc*_*beutel*_*masc*_ [teabag] the constituent nouns share their gender (masculine), which constitutes a gender match. In addition to semantic transparency, we investigated whether the first constituent's gender is activated during compound naming. If so, mismatch might produce processing costs, relative to same-gender targets. Such a pattern would corroborate morpheme-based processes at a level that codes syntactic word properties, such as the lemma level (see Marelli et al., 2012; for a unitary lemma view for complex words, see Levelt et al., 1999).

Our study thus investigates the lexical representation of compounds, as a function of their semantic transparency, (1) at the word-form (or lexeme) level, and (2) at the lemma level—by means of the gender match of a compound's constituents. Before providing the details of our study, we review theories of speech production and their predictions for (constituent) gender and semantic transparency effects in compound production. Next, we introduce the picture-word paradigm and its uses, and summarize the available evidence on morphological processing in production.

### The two-stage model of speech production

Models of speaking assume multiple levels of processing and representation, even for one-word utterances (Garrett, 1982; Dell, 1986; Levelt, 1989). A well-known representative, the two-stage model, assumes two separate lexical levels, lemmas, and lexemes (word forms) (Levelt, 1989; Levelt et al., 1999). Grammatical gender is stored at the lemma level, at which all syntactic properties of words are specified^1^. Information about a word's surface form, including its constituent morphemes and phonemes, is stored at the lexeme level. Thus, the lemma is an intermediate representation between semantic and morpho-phonological information, linking the semantic and grammatical features of a word.

When a picture is named by means of a German determiner-noun phrase (e.g., *das*_*neut*_ *Haus*_*neut*_[the house]), the following steps take place: (1) the picture activates its own concept as well as related concepts, which (2) leads to the activation of multiple lemmas. From these activated lemmas, the target lemma has to be selected, along with its grammatical features, including gender (neuter for *Haus*). The corresponding lexical-phonological information (determiner + noun → *das Haus*), stored at the word-form level, is activated by the selected lemma (see Roelofs, 1992; Jescheniak and Levelt, 1994; Indefrey and Levelt, 2004; Indefrey, 2011; for an interactive activation account, see Dell, 1986; Dell et al., 1997; for a model that does not assume a separate lemma level, see Caramazza, 1997).

According to Levelt et al. (1999), compounds are stored in a decomposed way at the word-form (lexeme) level, and the constituent morphemes are retrieved during speech production (for empirical evidence, see Lüttmann et al., 2011a; for a contrasting account, see Janssen et al., 2008, 2014). Furthermore, the model assumes holistic compound representations at the lemma level (“the single-lemma-multiple morpheme case,” pp. 12, Roelofs et al., 1998; Levelt et al., 1999). Thus, on the single-lemma account, grammatical features of the modifier, such as its grammatical gender, should not affect compound production. However, the single-lemma view has only rarely been tested (but see Lorenz et al., under review; Lüttmann et al., 2011a; Lorenz and Zwitserlood, 2014).

### The picture-word paradigm

A common tool to study the processes underlying speech production is the picture-word task (also called picture-word interference paradigm). In this task, pictures are presented together with written or spoken distractor words, and participants are instructed to name the pictures as quickly as possible, while ignoring the distractors. The relation of distractor to picture (name) affects naming latencies and accuracies in specific ways. For example, distractor words denoting concepts from the same semantic category (“categorical relatedness”) most often interfere with lexical retrieval and thus increase naming latencies, whereas phonologically related distractors facilitate picture naming. Morphological relatedness also induces facilitation, and to a larger degree than mere phonological overlap (Roelofs and Baayen, 2002; Dohmes et al., 2004).

Picture-word interference was also used to study effects of grammatical gender in speech production. In German or Dutch, which have different gender classes, picture naming in a gender-marked format (i.e., production of gender-marked determiner-noun or adjective-noun phrases) is delayed when (semantically unrelated) distractors differ from the target noun in gender, compared to gender-congruent distractors (e.g., Schriefers, 1993; La Heij et al., 1998; Schriefers and Teruel, 2000). Note that these effects are fickle in Romance languages (Miozzo and Caramazza, 1999; Foucart et al., 2010), and are not even consistent in Germanic languages (see Pechmann and Zerbst, 2004; Schiller, 2013; for published failures to replicate).

### The production of compounds: evidence for compositional processes

Most studies on compound processing in speech production point to morpheme-based representations at minimally one lexical level (Zwitserlood et al., 2002; Dohmes et al., 2004; Lüttmann et al., 2011a; Lensink et al., 2015). Whether this also holds for the production of semantically opaque compounds has not been tested because most studies manipulate the semantic transparency on the distractors, not on the targets for production (see below). Data from the implicit priming paradigm, examining the production of Dutch derived nouns, however, point to decomposition for opaque forms, too (Roelofs and Baayen, 2002).

An early study on German (Dohmes et al., 2004) used semantically transparent and opaque compound distractors, and simple nouns as targets for picture naming. Morphological facilitation occurred irrespective of the semantic transparency of the distractors (for similar data from a translation task, see Gumnior et al., 2006). Morphological effects in speech production were disentangled from purely phonological effects, because pseudo-complex, monomorphemic distractors (e.g., *Neurose* [neurosis]), as distractor to a picture of a rose) induced significantly less facilitation than compound distractors (e.g., *Buschrose* [bushrose]) (Dohmes et al., 2004; Köster and Schiller, 2008, for a replication with EEG; Verdonschot et al., 2012). Note that these studies investigated the interface between production (of simple words) and comprehension (of complex words), rather than compound production. This was different in a picture-word interference study by Lüttmann et al. (2011a) who used compounds as targets and distractors, which overlapped in the second morpheme (e.g., distractor: carpetbag, target: handbag). Picture naming was facilitated by morphologically related distractors, and effects were similar for semantically transparent and opaque distractors (e.g., distractor: scumbag, target: handbag). Note that the production of semantically opaque compounds/complex words was not investigated in this study. This was done by Roelofs (1996b) using complex words starting with a preposition (e.g., inlaw, uphill). He used the implicit priming paradigm, in which sets of words are produced within one trial (e.g., inlaw, input, insult vs. outlaw, uphill, input). Comparing trials with and without onset overlap, speech preparation effects were stronger when the overlap constituted a morpheme (as in byline, bypass, bylaw) rather than merely a syllable (bible, bypass, biker). Note that this effect held even when the sets included semantically opaque complex words (Roelofs and Baayen, 2002).

Another way to investigate the processes underlying compound production is to manipulate constituent and full-form frequency. Roelofs (1996a), with the implicit priming paradigm, reported morpheme-based frequency effects in Dutch compound production, and argued for morpheme-based representations of compounds (see also Bien et al., 2005; for evidence from aphasia, see Blanken, 2000). In contrast, Janssen et al. (2008) observed frequency effects^2^ for the compounds' full forms, not for constituent morphemes, which they interpreted as evidence for holistic representations of compounds at the word-form level (see also Bi et al., 2007; Janssen et al., 2014; for a contrasting interpretation, see Taft, 2004; Baayen et al., 2007).

Furthermore, evidence from aphasia mainly points to decomposed lexical form representations in speech production (Semenza et al., 1997, 2011; Blanken, 2000; Badecker, 2001; Lorenz and Zwitserlood, 2014; Lorenz et al., 2014). In favor of decomposition are specific difficulties in the naming of compounds compared to matched simple nouns, such as more errors with compounds than simple nouns, and constituent errors with compound targets in picture naming. In simplifications, for example, one constituent of the compound target is retrieved, while lexical access to the other constituent is blocked (e.g., “*butter and something*…” in response to *butterfly*). Thus, simplifications and other types of constituent errors in compound naming reflect sensitivity to the morphological structure of the target (e.g., Blanken, 2000; Lorenz et al., 2014).

In sum, the available evidence mainly points to morpheme-based storage and processing of compounds on at least one lexical level in production (but see Janssen et al., 2008, 2014). While most studies locate morphological effects at the word-form level, effects might also originate at the lemma level, or at both word-form and lemma level. As explicated below, this idea has hardly been tested until today. Moreover, it is still unresolved whether the semantic transparency of compounds has an impact at any point during their production.

### The production of compounds: single or multiple lemmas

According to the two-stage model (Levelt et al., 1999), syntactic features of a noun, including grammatical gender, are stored at the lemma level. Because single, holistic lemmas are assumed for compounds, retrieval of grammatical gender information should not differ between compounds and simple nouns, or between same-gender and different-gender noun-noun compounds. This model, therefore, predicts that the gender or other syntactic features of non-head (modifier) constituents should be irrelevant in compound production.

As mentioned earlier, individuals with aphasia show evidence for decomposed lemma representations of compounds. Data from agrammatic participants are particularly interesting in this respect because they typically suffer from word-category deficits for verbs, and usually, this deficit persists in the naming of verb-noun compounds (e.g., *Roll*_*V*_*schuh*_*N*_ [rolling shoe = roller skate]), whereas retrieval of noun-noun compounds, especially of the first (nominal) constituent, is better preserved (e.g., *Haus*_*N*_*schuh*_*N*_[house shoe = slipper]; Lorenz et al., 2014). A similar pattern was reported in an Italian word-reading study with a person with phonological dyslexia (Marelli et al., 2012; see also Mondini et al., 2004, 2005). According to current accounts, grammatical word-category deficits in aphasia result from a deficit in accessing lexical-syntactic features at the lemma level (Berndt et al., 1997a,b; Crepaldi et al., 2006, 2011; but see Rapp and Caramazza, 2002; Mätzig et al., 2009). These data thus point to decomposed lemma representations of compound nouns (Marelli et al., 2012).

Another way of studying compounds at the lemma level is to manipulate the gender match of their constituents. Some comprehension studies reported constituent-gender effects for noun-noun compounds (e.g., Köster et al., 2004; Jalbert et al., 2016, with EEG; see Meunier et al., 2008 for data from derived nouns). It is still unresolved, however, whether these effects also occur in production paradigms, such as picture naming. In a study with German aphasic participants, we compared picture-naming accuracy for same-gender and different-gender noun-noun compound targets (Lorenz and Zwitserlood, 2014). Participants were instructed to name pictures with determiner-compound noun phrases, and thus a gender-marked determiner had to be retrieved in addition to the compound target (e.g., *das*_*neut*_ *Vogelhaus*_*neut*_ [the birdhouse]). Different-gender compounds revealed no processing costs relative to same-gender compounds. One problem, however, is that the data might reflect a floor effect because the participants suffered from severe word-finding difficulties concerning retrieval of determiners and nouns.

In a production study on lemma representations of compounds with language-unimpaired speakers, Lüttmann et al. (2011a) also did not obtain any evidence for decomposed compound lemmas. Instead of manipulating gender, they examined semantic interference in picture naming with compounds as targets, using same-category distractors. Semantic interference is taken to reflect competition at lemma level (Roelofs, 1992; but see Mahon et al., 2007). Distractors were either semantic coordinates of the target compounds (distractor: suitcase; target: handbag), or related to only the first or the second constituent of compound targets, but not to the complete compound (distractor: foot, target: handbag). If the latter distractors induce interference, this would point to decomposed lemmas of compounds. Semantic interference was found when distractors were semantically related to the whole compound, not when distractors were merely related to a constituent. The authors thus concluded that compounds have holistic lemma representations. However, the targets in this experiment consisted of fully transparent, semi-transparent, and opaque compounds. If lemma representations of compounds are affected by semantic transparency, potential effects cannot be disentangled here.

In sum, the processes and representations involved in compound production are still a matter of debate. Whereas most evidence points to morpheme-based lexical representation, the impact of a compound's semantic transparency and its grammatical features in speaking is still unresolved. Although most evidence points to holistic lemma representations, the syntactic word-category data from patients might be problematic for this view, and alternative models have been proposed accordingly (Marelli et al., 2012).

### Aim of present study

Our study examines the lexical representation and processes involved in the production of determiner-compound noun phrases, using a picture-word interference paradigm. Taking the two-stage model (Levelt et al., 1999) as our working model, we investigated the lexical representation of compounds with regard to syntactic features (gender) at lemma level, and with regard to their morpho-phonological representation at word-form/lexeme level. Our first question is whether the semantic transparency of compound targets in any way affects their production, as predicted by dual-route accounts developed for comprehension. A second question concerns the representation and processing of compounds as a function of grammatical aspects of their constituents (here: grammatical gender) during speech production.

## Methods

### Outline of experiment

In a picture-word interference paradigm, participants produced determiner-compound noun phrases in German, a language that marks grammatical gender on the definite determiners of nouns. The semantic transparency and gender match of the target compounds was manipulated orthogonally in a factorial design. Thus, half of the compounds was semantically transparent (e.g., *Sektglas* [champagne glass]), the other half was opaque (e.g., *Löwenzahn* [lion+tooth = dandelion]). Within each transparency set, half of the targets differed with respect to their constituents' gender (different gender, e.g., *Sekt*_*masc*_*glas*_*neut*_), whereas the other half had constituents of the same gender (same gender; e.g., *Tee*_*masc*_*beutel*_*masc*_).

Three related distractor conditions were compared to one unrelated control condition. Related distractor nouns overlapped with the target in either the first or second constituent, or were merely gender-congruent with the target. Note that by rule, the second-constituent distractors were always gender-congruent with the compound, whereas first-constituent distractors were gender-incongruent with the target in case of different-gender compounds, and gender-congruent, in case of same-gender compounds. Control distractors were unrelated to the target compound with regard to semantic, morphological, phonological, and syntactic (gender) properties (see Table 1 for examples).

**Table 1:** **Distractor Conditions**.

	**Distractor Condition**	**Distractor**	**Target**
D1	Gender-congruent	Blatt_*neut*_ [leaf]	
D2	First constituent	Sekt_*masc*_ [sparkling wine]	
D3	Second constituent	Glas_*neut*_ [glass]	das_*neut*_ Sekt_*masc*_glas_*neut*_
	Unrelated control	Ziege_*fem*_ [goat]	[the champagne glass]

### Predictions

Specific predictions derived from the two-stage theory (Levelt et al., 1999) are as follows. The study taps into the representation of compounds at lemma level, where grammatical gender is stored, and into their representation at word-form level, where the constituent morphemes are represented. Given the assumption of single lemmas for compounds, the theory predicts no effects of the constituents' gender in compound production. Thus, matched subsets of same-gender and different-gender compounds should not differ in our behavioral measures (latencies and accuracies).

The two-stage model assumes morpheme-based (decomposed) word-form representations of compounds, and thus faster naming with morphologically overlapping distractors than with unrelated distractors (see Lüttmann et al., 2011a). Whether this effect is modulated by the semantic transparency of compound targets has not been tested before. If opaque compounds have a single word-form representation and transparent compounds are assembled from their constituent morphemes, as proposed by dual-route accounts, stronger facilitation for transparent than opaque compounds should be obtained. In addition, we expect to replicate the gender-congruency effect with compound targets, which has often been observed with monomorphemic targets (but see Pechmann and Zerbst, 2004). We predict longer naming latencies with gender-incongruent than gender-congruent distractors (e.g., Schriefers and Teruel, 2000; Schiller and Caramazza, 2003). Furthermore, in case of holistic compound lemmas, the gender-congruency effect induced by distractor nouns should not interact with gender match between the constituents of compound targets (same gender vs. different gender constituents).

In contrast, the multiple lemma representation account (Marelli et al., 2012) assumes that the lemmas of a compound's constituents are accessed in addition to a holistic compound lemma during compound production. The account therefore predicts processing costs in naming different-gender compared to same-gender compounds (see Köster et al., 2004, for data from comprehension). In naming different-gender compounds (with a gender-marked determiner), the first constituent's gender needs to be inhibited (e.g., different gender: *das*_*neut*_ *Sekt*_*masc*_*glas*_*neut*_ vs. same gender: *die*_*fem*_ *Luft*_*fem*_*pumpe*_*fem*_ [the air pump]). Therefore, naming should be slower and more error-prone for different gender than for same-gender compounds, and interactions with effects of gender congruency of distractor nouns are expected. Whether a multiple lemma representation account also holds for semantically opaque compounds has not been tested so far.

### Participants

Twenty native speakers of German participated in the experiment (13 women, mean age: 23.8 years; range: 19–37 years). All participants were students of the University of Münster and had normal or corrected to normal vision. They received course credit for their participation.

### Experimental materials

The experimental item set consisted of 40 noun-noun compounds and the corresponding object pictures (Hemera Photo Objects). In addition, 10 nominal compounds and 30 monomorphemic nouns were included as fillers (overall, *n* = 40 fillers). The semantic transparency and gender match of the experimental compounds was manipulated; half of the items was semantically transparent, the other half was opaque, and half of the items in each transparency group were different-gender compounds (e.g., *Vogel*_*masc*_*haus*_*neut*_ [birdhouse]), whereas the other half included same gender compounds (e.g., *Tee*_*masc*_*beutel*_*masc*_ [tea bag]).

Norms for semantic transparency were obtained by means of a rating with 70 native speakers of German (see Lorenz and Zwitserlood, 2014, for details). The semantic transparency of compounds was evaluated separately in relation to each constituent. For example, *Löwenzahn* [“lion+tooth” = dandelion] is fully opaque because neither “lion” nor “tooth” are related to the compound's meaning, whereas *Fliegenpilz* [“fly+mushroom” = fly agaric] or *Notenschlüssel* (“note+key” = clef) are partially opaque because only one constituent is semantically unrelated to the compound, whereas the semantic relation with the other constituent is transparent (opaque constituents are underlined; see also Zwitserlood, 1994; Libben et al., 2003). In the rating study, compounds and constituents were embedded in declarative sentences, such as “The meaning of *butter* is part of the meaning of *butterfly*.” The validity of the sentences was rated on a 6-point Likert scale with alternatives ranging from “the statement is completely correct” (point 6) to “the statement is completely incorrect” (point 1). For the purpose of this study, two subsets of depictable compound targets were used, closely matched for a number of factors, but differing in semantic transparency (see Table 2 for mean transparency values of transparent and opaque sets).

**Table 2:** **Mean semantic transparency values on the basis of a rating-study (6-point Likert skale; high values indicate high transparency, low values indicate low transparency)**.

	**Semantic transparency**	
	**Transparent**	**Opaque**
First Constituent	4.48 (0.59)	2.77 (1.17)
Second Constituent	5.07 (0.47)	2.61 (0.78)
mean	4.78 (0.53)	2.69 (0.98)

The semantically transparent subset included only fully transparent compounds, the opaque subset included 16 fully opaque compounds and four compounds with a semantically transparent modifier, but an opaque head constituent (e.g., *Notenschlüssel* [“note+key” = clef]) (see Table 2, for complete list of items see Table A1 (Appendix) in Supplementary Material). Each transparency group included 10 same gender and 10 different gender noun-noun compounds. Between same gender and different gender items and between transparent and opaque items, word-frequency (i.e., lemma frequency per Million) of the full form and the constituents (dlex database, Heister et al., 2011), word length (number of phonemes and syllables), number of lexical neighbors (Coltheart et al., 1977), and grammatical gender were matched (*p* > 0.05 each; see Tables A2, A3, Supplementary Material). Transparent and opaque subsets differed significantly on the transparency values of the whole word, and on the transparency values of the second constituents (see Table A2). The three genders masculine, feminine, and neuter occurred with similar proportions overall, and in the different subsets (see Tables A2, A3).

The unrelated condition was created by re-sorting pictures and distractors of the gender-congruent condition, creating gender-incongruent pairs that were also morphologically, semantically and formally (phonologically, orthographically) unrelated. The distractors of the two morphological conditions (1st and 2nd constituent of target) were matched according to word-frequency, length, and number of lexical neighbors (*p* > 0.1, all, see Table A4). The first- and second-constituent distractors were significantly more frequent than the unrelated controls (see Table A4 for details). Distractor frequency was included as a covariate into our statistical model to control for a potential impact of frequency differences of control distractors and constituent distractors (see below).

For the filler targets (*n* = 40), distractors were either (1) semantically related (*Posaune*_*fem*_ [trombone]), (2) gender congruent (*Puppe*_*fem*_ [doll]), or (3) unrelated (*Kissen*_*neut*_ [pillow]) to the target (*Trompete*_*fem*_ [trumpet]). In the unrelated condition, each filler target was presented twice, but with different distractors. Thus, all fillers and experimental targets were presented four times in the course of the experiment. The semantically related fillers were mostly gender-incongruent (85%) so that overall, gender congruency was balanced (51.9% gender congruent; 48.1% gender incongruent). In addition, the fillers served to decrease the proportion of morphologically related distractor-target pairs to approximately 30% overall (see Lüttmann et al., 2011a for a similar procedure).

### Procedure

All pictures were adjusted to a height of 300 pixel. Written distractors were presented in black, font Arial size 36 directly above the target object. As is common in speech production research, the target pictures were presented in each distractor condition, and were thus repeated within participants (e.g., Schiller and Caramazza, 2003; Dohmes et al., 2004; Lüttmann et al., 2011a). In this experiment, each participant saw each picture four times, each time with a different distractor. Overall, 320 target-distractor pairs were presented, and the target-distractor pairs were distributed across four lists using a Latin-square design. Each target appeared only once per list, with a different distractor on each list. At least 15 items intervened between repeated presentations of the same picture. Similarly, at least 15 items intervened between identical distractor words. Other than this, the targets were presented in randomized order, and each participant received a different order.

**Figure 1:**
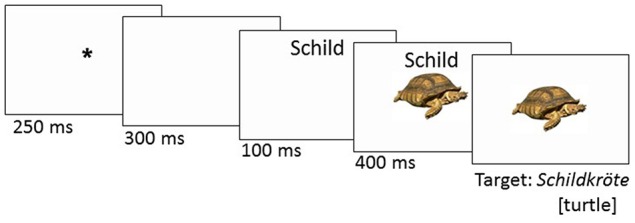
**Experimental design: Example from 1st constituent condition**.

In a familiarization phase prior to the experiment, participants saw all target pictures with their written names. They were instructed to use these words when naming the pictures in the experiment. Sixteen practice items preceded the test trials, with targets and distractors that differed from the experimental items. Participants were tested individually in a quiet room, sitting in front of a computer screen. They were instructed to name the pictures as quickly and accurately as possible. Pictures and distractors were presented against a white background. Each trial started with a fixation cross for 250 ms. A blank screen followed for 300 ms, after which the written distractor was presented for 500 ms. After 100 ms, the target picture appeared directly under the written distractor word (stimulus-onset asynchrony, SOA–100 ms)^3^. While the distractor word was presented slightly above the center (250 pixels above), the target pictures were presented in the center of the screen (see Figure 1). Participants were instructed to name the object depicted on each picture, and to ignore the distractor words. The experiment lasted for about 35 min. Compound naming latencies and response accuracies were registered.

The Presentation® software package was used to run the experiment (http://www.neurobs.com), and naming responses were recorded online. Naming latencies were measured manually after the experiment from the audio recordings of the participants' responses, using a PRAAT script (Boersma and Weenink, 2016). The expert who measured response latencies was blind with respect to experimental conditions. Participants produced determiner-noun phrases, and two different latency measures were determined: (1) the latency from picture onset to the speech onset of the determiner (analysis 1), and (2) the latency from picture onset to the speech onset of the noun (analysis 2). Because the two measures showed almost identical results, the data of analysis 1 are reported here, and results of analysis 2 are only reported if deviating from analysis 1. On the basis of the audio recordings, responses were coded as erroneous in cases of dysfluencies, of searching behavior, and of incorrect determiners and/or noun responses.

## Results

The data of one participant were excluded due to a high error rate for experimental targets (29% errors). The remaining 19 participants responded with a mean accuracy of 96% (range 87.5–100%). One compound target of the same gender, semantically opaque subset (Stimmgabel [tuning fork]) was excluded from the data set due to misclassification as a noun-noun compound. None of the other experimental targets had to be excluded since each target was named with an accuracy of at least 84%. Thus, data from 39 compounds and 19 participants were analyzed. Naming latencies greater than 2500 ms were discarded, and latencies deviating from a participant's and item's mean by more than 2 *SD* were considered as outliers and excluded from the RT analysis, resulting in a loss of 349 trials (11.8%). Mean naming latencies, effect sizes of the latency data, and percentages of correct responses are given in Table 3.

**Table 3:** **Mean naming latencies, SD (in parentheses), effect size (difference score, control - related), and percent correct as a function of distractor condition, semantic transparency, and gender match of constituents of compound targets**.

**Condition**	**Semantic transparency**					
	**Transparent**			**Opaque**		
	**M (SD)**	**Effect size**	**% correct (SD)**	**M (SD)**	**Effect size**	**% correct (SD)**
**GENDER-MATCH**						
D1: Gender-congruent	795 (171)	+36	96 (0.2)	836 (170)	−24	96 (0.2)
D2: First constituent	727 (142)	+104	99.5 (0.1)	763 (164)	+49	99 (0.1)
D3: Second constituent	743 (146)	+88	95 (0.2)	775 (166)	+37	98 (0.1)
Control	831 (185)		94 (0.2)	812 (161)		96 (0.2)
Mean	774 (167)		96 (0.2)	796 (167)		98 (0.2)
**GENDER-MISMATCH**						
D1: Gender-congruent	808 (150)	−19	94 (0.2)	834 (162)	−7	97 (0.2)
D2: First constituent	751 (152)	+38	94 (0.2)	752 (135)	+75	97 (0.2)
D3: Second constituent	764 (148)	+25	92 (0.3)	750 (153)	+77	98 (0.1)
Control	789 (155)		93 (0.3)	827 (160)		97 (0.2)
Mean	777 (153)		93 (0.3)	791 (158)		97 (0.2)

*Size of effect = control—related (D1, D2, or D3)*.

Naming accuracies and logarithmically transformed latencies were included as dependent variables in separate linear mixed models (LMM), using the lme4 package in R (version 1.1-6; Bates et al., 2014; see also Baayen et al., 2008). For accuracies, logit mixed-effects models (generalized linear mixed models, binominal family) were run (Jaeger, 2008). *P*-values were computed with the lmerTest package. Gender match (same vs. different gender of compound constituents) and distractor condition (D1: gender-congruent, D2: first constituent, D3: second constituent) were included as fixed factors; distractor frequency was included as a continuous variable. Although semantic transparency of our compounds was also varied in a factorial design, semantic transparency was included as a continuous variable, using mean transparency values of constituent 1 and 2 for each target (see Baayen, 2010; Marelli and Luzzatti, 2012). Sliding difference contrasts were set for gender match (same vs. different gender of compound constituents). For the related distractor conditions, simple contrasts, comparing the unrelated control condition with each of the related distractor conditions were used (D1: gender-congruent noun; D2: first constituent of target; D3: second constituent of target). The continuous variables (semantic transparency and distractor frequency) were centered.

A first full model revealed that distractor frequency did not contribute significantly. Model comparisons confirmed that distractor frequency did not contribute significantly to the goodness-of-fit of the models, so that distractor frequency was excluded (see Appendix for AIC and BIC values of full and reduced models of latency and accuracy data; Table A5). For the latency data, additional *post-hoc* models were run to assess the origin of significant interactions, and additional *post-hoc t*-tests were run, if necessary.

### Naming accuracies

A main effect of first-constituent distractors was obtained in the accuracy data (*z* = 2.803; *p* = 0.005) because fewer errors occurred for compounds paired with first-constituent distractors than with unrelated distractors (see Table 3). In contrast, the error rates for second-constituent or gender-congruent distractors did not differ from the unrelated distractors. Note that first-constituent distractors and picture names share the same onset, and first constituents are thus likely to be a better access cue than second constituents. Furthermore, first constituents might facilitate production at sub-lexical levels. Naming accuracy was neither affected by semantic transparency nor by gender match of the compound targets. However, semantic transparency interacted with gender match (*z* = −2.689, *p* = 0.007), showing that there were fewer errors with semantically transparent same-gender than different-gender compounds.

### Naming latencies

Table 4 shows the results of the LMM analysis for the naming latencies (analysis 1)^4^. The table includes the estimates, standard errors (*SE*), *t*-, and *p*-values for the main effects, and interactions. Distractor effects were analyzed for each condition (D1 = gender-congruent noun; D2 = 1st constituent; D3 = 2nd constituent) separately. Thus, a significant main effect of a related distractor condition reflects a significant difference from the unrelated control condition.

**Table 4:** **Results of LMM with subjects and items as random intercepts**.

		**Estimate**	***SE***	***t*-value**	**Pr(>|t|)**
**FIXED EFFECTS**					
(Intercept)		6.65	0.03	291.11	<0.001^***^
D1 (Gender-conguent distractor)		0.003	0.01	0.32	0.749
D2 (First-constituent distractor)		−0.09	0.01	−9.61	<0.001^***^
D3 (Second-constituent distractor)		−0.07	0.01	−8.11	<0.001^***^
GenderMatch within target		0.01	0.02	0.49	0.625
Transparency (centered)		−0.01	0.01	−1.81	0.078^(*)^
D1xGenderMatchxTransparency		0.03	0.02	2.19	0.029^*^
D2xGenderMatchxTransparency		0.04	0.02	2.55	0.011^*^
D3xGenderMatchxTransparency		0.04	0.02	2.77	0.006^**^
**RANDOM EFFECTS**					
Groups	*SD*	Log likelihood:			
Target	0.04	972			
Subject	0.09	REML deviance:			
Residual	0.16	−1944			

*Gender Match = same vs. different gender of constituent nouns within compounds*.

*All results for main effects are reported. Interactions are only reported if significant*.

(*) *p < 0.1*;

* *p < 0.05*;

** *p < 0.01*;

*** *p < 0.001*.

Significant main effects were obtained for the two morphological distractors (see condition D2 and D3, Table 4). Participants' compound naming was facilitated by first and second constituent distractors, and their overall effect sizes (unrelated—related) did not differ significantly (68 ms for first constituent, and 58 ms for second constituent; paired *t*-test, first vs. second constituent, all *p* > 0.1). In contrast, gender congruency between distractor and compound target (D1) did not affect overall naming latencies (see Table 4).

Similarly, gender match, that is, whether the constituent nouns of the targets had the same or different gender, did not significantly affect overall naming latencies. However, gender congruency between distractor and compound target interacted with gender match of the compound's constituents, and with its semantic transparency. Similarly, for the morphological conditions, three-way interactions with gender match and semantic transparency were obtained. To shed light on these interactions, *post-hoc* models were run for semantically transparent and opaque targets separately. These models confirmed a different pattern as a function of the semantic transparency of the compound targets. For transparent targets, the effects of the distractor conditions differed depending on whether the constituents of the target had the same or different gender, as indicated by significant interactions (see Table 5). In contrast, for opaque compound targets, none of these interactions reached significance (see Table 6), showing a similar pattern for same-gender and different-gender compound targets.

**Table 5:** ***Post-hoc* model with subset of items: semantically transparent targets**.

**Fixed effects**		**Estimate**	***SE***	***t*-value**	**Pr(>|*t*|)**
(Intercept)		6.64	0.03	273.76	<0.001^***^
D1 (Gender-congruent distractor)		−0.01	0.01	−0.80	0.442
D2 (First-constituent distractor)		−0.01	0.01	−7.29	<0.001^***^
D3 (Second-constituent distractor)		−0.07	0.01	−5.64	<0.001^***^
GenderMatch within target		0.01	0.02	0.60	0.555
D1xGenderMatch		0.07	0.03	2.70	0.007^**^
D2xGenderMatch		0.07	0.03	2.81	0.005^**^
D3xGenderMatch		0.06	0.03	2.41	0.016^*^
Random effects		Log likelihood:			
Groups	*SD*	488			
Target	0.04	REML deviance:			
Subject	0.1	−976			
Residual	0.16				

*GenderMatch = same vs. different gender of constituent nouns of targets. Results of LMM with subjects and items as random intercepts. All results for main effects are reported. Interactions are only reported if significant*.

* *p < 0.05*;

** *p < 0.01*;

*** *p < 0.001*.

**Table 6:** ***Post-hoc* model with subset of items: semantically opaque targets**.

**Fixed effects**		**Estimate**	***SE***	***t*-value**	**Pr(>|*t*|)**
(Intercept)		6.66	0.03	271.14	<0.001^***^
D1 (Gender-congruent distractor)		0.02	0.01	1.52	0.128
D2 (First-constituent distractor)		−0.08	0.01	−6.10	<0.001^***^
D3 (Second-constituent distractor)		−0.07	0.01	−5.60	<0.001^***^
GenderMatch within target		−0.004	0.02	−0.15	0.88
Random effects		Log likelihood:			
Groups	*SD*	474			
Target	0.09	REML deviance:			
Subject	0.05	−948			
Residual	0.16				

Results of LMM with subjects and items as random intercepts. All results for main effects are reported. Interactions are only reported if significant. ^*^p < 0.05;^**^p < 0.01;

*** *p < 0.001*.

Additional *post-hoc t*-tests revealed that participants indeed showed a facilitating gender-congruency effect (36 ms), that is, faster naming with gender-congruent than—incongruent distractors. This effect, however, was only obtained with semantically transparent compound targets consisting of two constituents of the same gender (transparent, same-gender targets: gender-congruent vs. gender-incongruent condition, *t* = −2.46; *p* = 0.014). For transparent targets with gender-mismatching constituents (different-gender targets) no significant gender-congruency effect was obtained (*t* = 1.33; *p* = 0.182). Similarly, for opaque targets no significant gender-congruency effect was present (see Table 6).

Furthermore, for semantically transparent targets, both morphological conditions interacted with gender-match of the compound's constituents, as morphological effects turned out to be stronger with same-gender than different-gender targets. In *post-hoc t*-tests, this difference reached significance for the first-constituent distractor [*t*_(18)_ = 2.26, *p* = 0.039], but not for the second-constituent distractor [*t*_(18)_ = 1.62, *p* = 0.122]. For opaque targets, these interactions did not reach significance, indicating a similar pattern with same-gender and different-gender targets, while significant main effects of both morphological conditions were obtained (see Table 6).

Importantly, the morphological conditions produced significant facilitation within each subset of items as confirmed by separate *t*-tests for the different subsets of items in a nested *post-hoc* model (same-gender and different-gender, semantically transparent and opaque targets all *ps* < 0.03; see Tables 5, 6; and Table A6). Furthermore, no two-way interactions of transparency with any of the distractor conditions were obtained. Thus, collapsed over gender match, the effects of gender congruency, and of morphological overlap (first and second constituent) were comparable for semantically transparent and opaque targets (see also Tables 3, 4).

Note that in analysis 2 (latencies until noun response), the overall pattern was similar. The three-way interaction of gender congruency, semantic transparency, and gender match, however, was not significant any longer, but there was a significant two-way interaction of gender congruency and gender match (estimate = 0.04, *t* = 2.24, *p* = 0.025). Collapsed over transparency, a nested *post-hoc* model revealed inhibition for different-gender targets (estimate = 0.03; *t* = 2.38; *p* = 0.017), but no significant effects for same-gender targets (estimate = −0.01; *t* = −0.75; *p* = 0.453).

## General discussion

In a picture-word interference paradigm participants produced determiner-compound noun phrases in response to object pictures. Noun-noun compound targets varied in semantic transparency and gender match, that is, half of the compounds was semantically transparent and the other half was opaque, and in half of the compounds within each transparency group, the compounds' constituents had the same grammatical gender, while in the other half the constituent nouns differed in gender. Effects of three related distractors (gender-congruent noun; first constituent of target; second constituent of target) were assessed against one unrelated distractor condition, and naming latencies and accuracies were measured. Our participants produced determiner+noun-noun compound phrases (e.g., *der Teebeutel*, the teabag, *das Sektglas*, the champagne glass), and thus—in addition to the compound noun itself—its grammatical gender had to be selected, to retrieve the corresponding gender-marked determiner (*der*_*masc*_*, die*_*fem*_, and *das*_*neut*_ [the]).

In a nutshell, we observed the following effects in the naming latencies. First and foremost, strong morphological facilitation was obtained, that is, naming latencies were significantly reduced when either the first or second constituents of compound targets were presented as distractors, relative to unrelated control distractors. Overall, the two constituents induced similar effects, and overall, facilitation was comparable for semantically transparent and opaque compound targets. Next, we observed no main effect of gender congruency, that is, naming latencies were comparable with gender-congruent and -incongruent distractors. In addition, gender match of the compound targets, that is, whether a compound consists of two nouns of the same gender or of different gender, had no main effect. However, interactions of gender match and semantic transparency with the different distractor conditions indicate that the gender of a compound's constituents and gender congruency of distractor and target did affect compound naming. Below, we go into the details of these interactions and discuss the relevance of the observed data pattern for the lexical representation and processing of compounds with regard to grammatical gender and morphological form in production, and the consequences for the models summarized in the introduction.

### Morphological effects in compound production

Morphological facilitation in speech production (over and above effects of form similarity) is assumed to reflect decomposed lexical representations (e.g., Zwitserlood et al., 2000, 2002; Roelofs and Baayen, 2002; Gumnior et al., 2006; Lüttmann et al., 2011a). We observed strong morphological facilitation, that is, naming a picture with a compound target was faster in the presence of morphologically overlapping distractors than of unrelated distractors. Thus, the data point to morpheme representations in the production lexicon. For the following reasons, we think it is unlikely that the morphological effects were due to pure phonological and/or semantic overlap, such as predicted by holistic lexical representation models (e.g., Janssen et al., 2008) or by network theories (e.g., Plaut and Gonnerman, 2000; Baayen et al., 2013). First, morphological effects have been disentangled from phonological effects in earlier studies (for evidence from the immediate picture-word task, see Dohmes et al., 2004; for evidence from a long-lag word-picture paradigm, see Zwitserlood et al., 2002; Köster and Schiller, 2008; for evidence from the implicit priming paradigm, see Roelofs, 1996b). Second, morphological facilitation effects on picture naming latencies were comparable for the first and second constituent. If effects were due to phonological overlap only, significantly stronger effects would have been expected for word-initial than word-final overlap, for the SOA used here (e.g., Meyer and Schriefers, 1991). The numerical advantage (10 ms) for first-constituent distractors might point to an additional sub-lexical contribution, but again, effects of the first and second constituents did not differ statistically.

Importantly, we can also exclude that the morphological effects were driven by the semantic relatedness between distractor and target *per se*, because comparable effects were obtained for semantically transparent and opaque compound targets (e.g., *Glas*_*neut*_ [glass] → *Sektglas*_*neut*_ [champagne glass] vs. *Zahn*_*masc*_ [tooth] → *Löwenzahn*_*masc*_ [lion+tooth] = [dandelion]). Thus, the semantic relatedness between distractor and compound target did not contribute to the effect sizes of the morphological conditions. Note that similar effects were found for transparent and opaque distractors (e.g., Dohmes et al., 2004; Köster and Schiller, 2008), which is good evidence for morphological parsing in comprehension, but does not say much about semantic and morphological processing during the actual production of complex words. But our current data do speak to this, and the effects support a decomposed representation, in terms of constituent morphemes, for compounds at a level of representation that is immune to the semantic relation between constituents and the compound as a whole. After Roelofs and Baayen (2002), who used implicit priming with semantically opaque derived words, this is the first evidence that semantic transparency does not influence morphological composition during speaking. The constituents needed for this morphological assembly would be stored at the word-form level, in the two-stage model (Levelt et al., 1999; for similar findings and conclusions, see Roelofs, 1996a,b; Roelofs and Baayen, 2002; Lüttmann et al., 2011a).

A potential point of critique is that the participants might have used an artificial morpheme-based strategy in compound naming. To counteract such strategic processes, morphological overlap between distractor and target was reduced to 30% of trials by including filler nouns, which were combined with morphologically non-overlapping distractors only. Furthermore, of 40 filler nouns, 30 targets were not morphologically complex. Therefore, a strategic explanation of the morphological effects is unlikely. Importantly, our evidence from compound production—observed with the picture-word task—fits with results from completely different paradigms that also revealed morpheme-based storage and processing of morphologically complex words in speech production (e.g., Roelofs, 1996a,b; Roelofs and Baayen, 2002; Bien et al., 2005; Köster and Schiller, 2008; Lorenz et al., 2014; Lensink et al., 2015; but see Janssen et al., 2008, 2014).

### Grammatical-gender effects in compound production

Next to morphological complexity, we investigated the representation and processing of compound targets at the lexical-syntactic (lemma) level during speech production. To do this, we included grammatical gender as a variable, both by manipulation of the gender match between the constituents of noun-noun compounds, and of a distractor that was semantically and morphologically unrelated, but gender-congruent to the target compound. Our participants produced noun phrases with definite determiners, which necessitates access to the compound's gender. Neither gender match of the targets' constituents, nor gender congruency of the distractors induced main effects. Note that effects of gender-congruent distractors are not robust in picture-word studies. Although there is published evidence for such effects in Germanic languages (see Jescheniak et al., 2014, for an overview), there are also published reports of failures to replicate (e.g., Pechmann and Zerbst, 2004; Schiller, 2013). It might be that we did not use the most appropriate SOA to obtain overall gender-congruency effects, but we did observe interactions that showed an impact of the constituents' gender and of gender congruency of distractors during noun-noun compound production.

According to the two-stage theory of speech production, compounds have holistic lemma representations, and morpheme-based form representations (e.g., Levelt et al., 1999). Following this, gender of the modifier constituent of noun-noun compound targets should not affect compound production. The absence of a main effect of gender match of the constituent nouns is in line with this view (for similar evidence from aphasia, see Lorenz and Zwitserlood, 2014; see also Lorenz et al., under review).

However, a significant three-way interaction of gender congruency with gender match of constituents and with the semantic transparency of compound targets was present. There was indeed an effect of gender-congruent distractors, but only for a subset of items: Only for transparent compounds with constituents that share their gender, such as *Tee*_*masc*_*beutel*_*masc*_ [tea bag] a significant (facilitating) gender-congruency effect was obtained, similar to the pattern usually observed with monomorphemic targets (e.g., Schiller and Caramazza, 2003). In contrast, gender congruency did not produce any significant effects with transparent compounds consisting of different gender constituents (*Sekt*_*masc*_*glas*_*neut*_ [champagne glass]), nor with opaque compound targets (e.g., *Löwen*_*masc*_*zahn*_*masc*_ [lion + tooth = dandelion]; *Esels*_*masc*_*ohr*_*neut*_[donkey+ear = dog-ear] (folded corner of a book page).

Moreover, in the morphological distractor conditions (first and second constituent of target), we also observed interactions of gender match of the constituents and semantic transparency. There was morphological facilitation in all cases, but only in case of transparent compound targets, the production of determiner + compound noun phrases (e.g., *der*_*masc*_ *Tee*_*masc*_*beutel*_*masc*_, [the teabag], paired with “tee” or “beutel” as distractor) was facilitated more when constituents had the same gender than when their gender differed.

One explanation might be that the representation of compounds at lemma level is affected by their semantic transparency. Semantically transparent compounds might have decomposed representations at lemma level, as assumed by the multiple lemma representation account (Marelli et al., 2012). In that case, there would be a match between the gender of the distractor, the first and the second constituent, which would have boosted access to the relevant determiner in case of same gender compounds. With this reasoning, opaque compounds would not possess multiple (decomposed) lemmas, because no significant interaction of gender match with gender congruency, nor enhanced morphological priming for gender-match targets were obtained here. But note that we did not observe any gender-congruency effects with different-gender transparent compound targets (e.g., *Sekt*_*masc*_*glas*_*neut*_ [champagne glass]). Insignificant inhibition was obtained here, and this inhibition effect turned out to be significant in our second latency measure (naming latency from noun onset). Thus, in case of different-gender targets, the modifier's gender was clearly accessed, which is also in line with a multiple-lemma representation account, and this mismatching gender information seemed to counteract the effects of a gender-congruent distractor noun, eliminating facilitation.

The question still remains why we did not observe any gender-congruency effects with opaque compounds. Studies with monomorphemic targets for German (Schriefers and Teruel, 2000; Schiller and Caramazza, 2003) showed effects of gender congruency, but note that other multi-experiment studies could not replicate these effects (Pechmann and Zerbst, 2004). Apparently, with our SOA and materials, the activation of only two lemmas with the same gender (opaque compound and distractor) was not strong enough to induce a congruency effect, but activation of four lemmas (transparent compound, first and second constituent, congruent distractor) sufficed to boost access to the determiner. To sum up, our data are in line with the multiple lemma representation account for semantically transparent, but not for opaque compound targets.

There is an alternative explanation, however, that also accounts for the data observed here. The two-stage model (Levelt et al., 1999) assumes single compound lemmas for semantically transparent and opaque compounds alike. But from the point of view of the percolation of semantic activation, from picture to concepts to the lexicon, it might be plausible to assume a difference as a function of semantic transparency. Let us assume that both types of compound have their own compound lemma, which is activated by the concept depicted in the picture (tea bag, or dandelion). In the case of a transparent compound, the concept “tea bag” would activate two semantically close additional concepts, “tea,” and “bag,” and their corresponding lemmas. But this would not happen for opaque compounds because the constituents of opaque compounds are not semantically related to the whole word (e.g., *Löwe* [lion] and *Zahn* [tooth] in *Löwenzahn* [dandelion]; see also Lorenz et al., 2014). Under the rather straightforward assumption of feed-forward activation from semantics to the lexicon (which is part of any speech production model), there would be three lemmas active in the case of transparent compounds, and only one for opaque compounds, in addition to other semantically related nouns, which are likely to be co-activated at lemma level (e.g., Roelofs, 1992). If all three lemmas are connected to the same gender—as is the case for same-gender transparent compounds—this might result in a clear boost of activation for the relevant determiner. This boost would even be stronger when a gender-congruent distractor is added—as is the case for the gender congruent, but otherwise unrelated distractor, as well as for both morphological distractors. This explanation is more parsimonious—applying Occam's razor—because it does not necessitate different representations for semantically transparent and opaque compounds, nor a hierarchical, multiple-lemma structure (Marelli et al., 2012). At the same time it relies on the co-activation of constituents and compound at lemma level (see Janssen and Caramazza, 2003, for a similar explanation for stem gender effects in the production of Dutch diminutive nouns).

To sum up, while our data are in line with the multiple lemma representation account for semantically transparent compounds (Marelli et al., 2012), an alternative, more parsimonious account (with holistic lemma representations), can also explain the results. Different co-activation patterns of constituents and whole word as a function of the semantic transparency of the target, originating from the conceptual level, can also account for the different impact of gender match and gender congruency in compound noun-phrase production. Note that—in contrast to the effects concerning gender congruency and gender match—overall morphological effects were substantive and did not differ between semantically transparent and opaque targets. Furthermore, first and second constituent distractors had a similar impact on compound naming, pointing to morpheme-based storage of compounds at the word-form level (Levelt et al., 1999). These effects corroborate the view that morphologically complex words are composed from their constituent morphemes during speaking.

## Conclusion

Our study is the first that manipulated the semantic transparency and grammatical gender of noun-noun compound targets in a picture-naming task. The data support lexical morpheme representations at the word-form level, which are unaffected by the semantic transparency of compound targets (Levelt et al., 1999). Furthermore, the data are compatible with multiple lemma representations for transparent, but not for opaque compounds. However, the more parsimonious account of holistic compound lemmas can also explain the data, because co-activation patterns of constituents and full forms are likely to differ for transparent and opaque targets. The study clearly shows that—in case of compound production—the possibility of co-activation of constituents and full forms, driven by the conceptual level, should be considered as a viable source of constituent-specific effects.

## Ethics statement

This study was carried out in accordance with the recommendations of ethical guidelines of the Institute for Psychology, Westfälische Wilhelms-Universität, Münster, Germany. All participants gave written informed consent in accordance with the Declaration of Helsinki.

## Author contributions

Both AL and PZ contributed to the experimental paradigm used here and the interpretation of the data. AL prepared the materials and analyzed the data. Both authors were involved in writing up the paper and both authors finally approved the version to be published.

## Funding

AL was supported by the German Research Council (DFG, LO 2182/1-1).

### Conflict of interest statement

The authors declare that the research was conducted in the absence of any commercial or financial relationships that could be construed as a potential conflict of interest.

## Footnotes

^1^Another name for the lemma level is lexical-syntactic level (e.g., Biran and Friedmann, 2012; Nickels et al., 2015).

^2^Janssen et al. (2008) employed word familiarity ratings instead of using word frequency measures.

^3^The SOA of −100 ms was chosen because morphological effects are reliably obtained with this timing of distractor and picture presentation (Dohmes et al., 2004; Lüttmann et al., 2011a). Note that gender congruency effects were sometimes observed with different SOAs (e.g., SOA 0 ms in (Schiller and Caramazza, 2003); SOA 75–150 ms in (Schriefers and Teruel, 2000)).

^4^We made sure that our model allowed for an adequate fit of the data based on the distribution of residuals (see Baayen, 2008).
