# Supplementary material for: Semantically Transparent and Opaque Compounds in German Noun-Phrase Production: Evidence for Morphemes in Speaking
Source: Front Psychol. 2016 Dec 27;7:1943. doi: 10.3389/fpsyg.2016.01943 (PMC5186785; doi:10.3389/fpsyg.2016.01943)
Supplement: Supplementary file 1 [file DataSheet1.pdf]

Appendix (Lorenz & Zwitserlood, 2016, Frontiers in Psychology)

Table A1: Experimental compound targets

Target	English translation	Trans-parency	1st const, transp	2nd const, transp	gender, compound	1st const, gender	gender-match
Luftschlange	air+snake = paper streamer	O	2.44	2.68	feminine	feminine	gm
Milchstraße	milk+street = milky way	O	1.44	2.79	feminine	feminine	gm
Löwenzahn	lion+tooth= dandelion	O	1.71	1.47	masculine	masculine	gm
Stammbaum	clan+tree= family tree	O	3.18	3.12	masculine	masculine	gm
Wintergarten	winter+garden	O	2.53	3.08	masculine	masculine	gm
Zollstock	inch+stick = inch rule	O	2.44	3.74	masculine	masculine	gm
Feuerzeug	fire+thing= lighter	O	5.06	1.68	neuter	neuter	gm
Hühnerauge	chicken+eye= clavus	O	1.26	1.59	neuter	neuter	gm
Meerschwein	sea+pig= guinea pig	O	1.41	2.26	neuter	neuter	gm
Armbrust	arm+chest= crossbow	O	2.76	2.26	feminine	masculine	gmm
Schildkröte	buckler+toad= tortoise	O	2.71	2.41	feminine	neuter	gmm
Wunderkerze	wonder+candle= sparkler	O	2	4.24	feminine	neuter	gmm
Handschuh	hand+shoe = glove	O	5.26	2.47	masculine	feminine	gmm
Notenschlüssel	note+key= clef	O	4.21	2.18	masculine	feminine	gmm
Fleischwolf	meat+wolve= meat chopper	O	4.38	1.44	masculine	neuter	gmm
Pferdeschwanz	horse+tail= pony tail	O	2.71	3.32	masculine	neuter	gmm
Nudelholz	noodle+wood= rolling pin	O	3.15	3.41	neuter	feminine	gmm
Eselsohr	donkey+ear= dog-ear	O	1.68	2.24	neuter	masculine	gmm
Raumschiff	space+ship	O	2.21	3.47	neuter	masculine	gmm
Postkarte	post+card	T	5.12	4.76	feminine	feminine	gm
Seifenblase	soap+bubble	T	4.06	4.29	feminine	feminine	gm
Taschenuhr	pocket+watch	T	3.85	5.68	feminine	feminine	gm
Briefkasten	letter+box = mail box	T	5.2	4.888	masculine	masculine	gm
Regenschirm	rain+ screen = umbrella	T	5.12	5.268	masculine	masculine	gm
Teebeutel	tea+bag	T	5.32	4.388	masculine	masculine	gm
Fotoalbum	photo+album	T	5.44	5.418	neuter	neuter	gm
Kinderbett	child+bed = crib	T	4.56	5.478	neuter	neuter	gm
Segelboot	sail+boat	T	5.38	5.53	neuter	neuter	gm

Appendix (Lorenz & Zwitserlood, 2016, Frontiers in Psychology)

Telefonbuch	telephone+book	T	4.03	5.12	neuter	neuter	gm
Hundehütte	dog+hut = doghouse	T	4.94	4.62	feminine	masculine	gmm
Mülltonne	rubbish+bin = trash can	T	5.53	5.47	feminine	masculine	gmm
Silbermünze	silver+coin	T	5.06	5.29	feminine	neuter	gmm
Windmühle	wind+mill	T	4.21	4.56	feminine	masculine	gmm
Angelhaken	fishing rod + hook = fish hook	T	5.35	4.76	masculine	feminine	gmm
Kugelfisch	sphere+fish = puffer fish	T	3.56	5.62	masculine	feminine	gmm
Ledermantel	leather+coat	T	5.29	5.56	masculine	neuter	gmm
Spinnennetz	spider+net	T	4.82	4.88	neuter	feminine	gmm
Halsband	throat+ribbon = necklace	T	4.82	4.26	neuter	masculine	gmm
Sektglas	champagne+glas	T	5.06	5.59	neuter	masculine	gmm

Note. Gm = gender match; gmm = gender mismatch; O = opaque, T = transparent

Table A2: Matching of transparent versus opaque compound targets according to linguistic factors. Mean values (standard deviation) are reported for transparent versus opaque subsets; for grammatical gender of the targets (masculine, feminine, neuter) absolute numbers and percentages are reported.

	Transparent (n=20)	Opaque (n=20)	*t*-value; *p*-value
Transparency, compound	4.9 (.4)	2.7 (.6)	13.7 < .001***
Transparency, constituent 1	4.8 (.6)	2.8 (1.2)	7.09 .537
Transparency, constituent 2	5.1 (.5)	2.6 (.8)	12.01 < .001***
Nb of letters	9.6 (1.0)	10.3 (1.6)	-1.04 .304
Syllables	3.3 (.6)	2.9 (.8)	1.82 .076
Letters, constituent 1	4.6 (1.0)	5.0 (1.4)	-.91 .364
Letters, constituent 2	4.8 (.8)	5.3 (1.5)	-1.29 .206
Stem frequency,* constituent 1	50.8 (110.2)	81.9 (119.9)	-.85 .399
Lexical neighbours, constituent 1	11.9 (7.0)	9.9 (6.5)	.93 .356
Stem frequency, constituent 2	46.6 (57.8)	59.2 (87.1)	-.54 .595
Lexical neighbours, constituent 2	11.5 (5.3)	8.4 (5.5)	1.83 .075
Stem frequency, compound	1.1 (1.1)	1.3 (2.4)	-.48 .635
Lexical neighbours**, compound	.3 (.5)	.2 (.4)	.62 .537
Grammatical gender of target			
Masculine	6 (30%)	8 (40%)	
Feminine	7 (35%)	6 (30%)	
Neuter	7 (35%)	6 (30%)	

*stem frequency = lemma frequency, normalised values (dlex DB, Heister et al., 2011)
** number of lexical neighbours (normalised) according to Coltheart et al., 1977

Table A3: Matching of same-gender and different-gender targets according to linguistic factors. Mean values (standard deviation) are reported for subsets of same- versus different-gender targets. For grammatical gender of the targets (masculine, feminine, neuter) absolute numbers and percentages are reported.

	Same gender (n=20)	Different gender (n=20)	*t*-value *p*-value
Transparency, constituent 1	3.6 (1.51)	4.0 (1.3)	-.83 .412
Transparency, constituent 2	3.8 (1.47)	3.9 (1.4)	-.28 .779
Nb of letters	10.1 (1.1)	10.1 (1.6)	.11 .91
Syllables	3.1 (.6)	3.0 (.8)	.44 .664
Letters, constituent 1	4.7 (1.0)	4.9 (1.4)	-.65 .520
Letters, constituent 2	5.1 (1.2)	5.1 (1.3)	.00 1.000
Stem frequency, constituent 1	72.6 (114.8)	60.1 (117.4)	.34 .736
Lexical neighbours, constituent 1	10.6 (6.7)	11.2 (6.9)	-.31 .76
Stem frequency, constituent 2	72.8 (97.9)	33.0 (24.1)	1.77 .085
Lexical neighbours, constituent 2	10.7 (6.2)	9.1 (4.8)	.90 .374
Stem frequency, compound	1.2 (1.1)	1.2 (2.4)	-.03 .975
Lexical neighbours, compound	0.3 (0.4)	0.2 (0.4)	.62 .537
Grammatical gender of target			
Masculine	7 (35%)	7 (35%)	
Feminine	6 (30%)	7 (35%)	
Neuter	7 (35%)	6 (30%)	

Table A4. Matching of distractor conditions (control, gender congruent, constituent 1 and 2): Mean values of linguistic factors (standard deviation), and *t*-values

Linguistic factors	1	2	3	independent *t*-test, 2-tailed		
	Control /gender congruent	Const. 1	Const. 2	t-value, 1 vs. 2	t-value, 1 vs. 3	t-value, 2 vs. 3
letters	4.6 (.9)	4.8 (.9)	5.1 (1.4)	-.935	-2.109*	-1.282
syllables	1-6 (.5)	1.5 (.6)	1.5 (.5)	.202	.422	.211
stem frequency	26.6 (36.3)	66.4 (114.8)	52.9 (73.2)	-2.088*	-2.033*	.626
lexical neighbours	11.1 (7.3)	10.9 (6.7)	9.9 (5.6)	.135	.827	.721

*= $p < .05$

Table A5. Model comparisons for naming latencies, and accuracies

	Models	df	AIC	BIC	logLik	deviance	Chisq/ p-value
Response latencies	final	19	-1906.1	-1794.6	972.04	-1944.1	15.6/ 0.2714
	full	32	-1895.7	-1707.9	979.84	-1959.7	

Table A6: Post-hoc nested LMM: Effects of the different distractor conditions (D1, D2, and D3) are reported for subsets of items.

Fixed effects:	Estimate	*SE*	*t*-value	Pr(>|t|)
(Intercept)	6.65	0.02	290.63	< 0.001***
g-match/transp: D1	-0.04	0.02	-2.46	0.014*
g-match/opaque: D2	-0.07	0.02	-3.57	<0.001***
g-mismatch/opaque: D2	-0.08	0.02	-4.91	<0.001***
g-match/transparent: D2	-0.01	0.02	-7.17	<0.001***
g-mismatch/transparent: D2	-0.06	0.02	-3.16	0.002**
g-match/opaque: D3	-0.05	0.02	-2.55	0.01*
g-mismatch/opaque: D3	-0.09	0.02	-5.25	<0.001***
g-match/transparent: D3	-0.1	0.02	-5.73	<0.001***
g-mismatch/transparent: D3	-0.04	0.02	-2.25	0.025*

Random effects

Groups	*SD*	
		Log likelihood: 972
Target	0.04	REML deviance: -1945
Subject	0.09	
Residual	0.16	

Note: D1 = gender-congruent distractor; D2 = First-constituent distractor; D3= Second-constituent distractor. Effects are only reported if significant.
Gender-match and semantic transparency were included as dichotomous factors.
